# Virus infection elevates transcriptional activity of miR164a promoter in plants

**DOI:** 10.1186/1471-2229-9-152

**Published:** 2009-12-30

**Authors:** Ariel A Bazzini, Natalia I Almasia, Carlos A Manacorda, Vanesa C Mongelli, Gabriela Conti, Guillermo A Maroniche, María C Rodriguez, Ana J Distéfano, H Esteban Hopp, Mariana del Vas, Sebastian Asurmendi

**Affiliations:** 1 Instituto de Biotecnología, CICVyA, INTA Castelar, Dr. N. Repetto y Los Reseros s/n, CP 1686 Hurlingham, Buenos Aires, Argentina; 2 Facultad de Ciencias Exactas y Naturales, Ciudad Universitaria, Buenos Aires, Argentina; 3 Consejo Nacional de Investigaciones Científicas y Técnicas (CONICET,) Buenos Aires, Argentina

## Abstract

**Background:**

Micro RNAs (miRs) constitute a large group of endogenous small RNAs that have crucial roles in many important plant functions. Virus infection and transgenic expression of viral proteins alter accumulation and activity of miRs and so far, most of the published evidence involves post-transcriptional regulations.

**Results:**

Using transgenic plants expressing a reporter gene under the promoter region of a characterized miR (P-miR164a), we monitored the reporter gene expression in different tissues and during *Arabidopsis *development. Strong expression was detected in both vascular tissues and hydathodes. P-miR164a activity was developmentally regulated in plants with a maximum expression at stages 1.12 to 5.1 (according to Boyes, 2001) along the transition from vegetative to reproductive growth. Upon quantification of P-miR164a-derived GUS activity after *Tobacco mosaic virus *Cg or *Oilseed rape mosaic virus *(ORMV) infection and after hormone treatments, we demonstrated that ORMV and gibberellic acid elevated P-miR164a activity. Accordingly, total mature miR164, precursor of miR164a and CUC1 mRNA (a miR164 target) levels increased after virus infection and interestingly the most severe virus (ORMV) produced the strongest promoter induction.

**Conclusion:**

This work shows for the first time that the alteration of miR pathways produced by viral infections possesses a transcriptional component. In addition, the degree of miR alteration correlates with virus severity since a more severe virus produces a stronger P-miR164a induction.

## Background

Small RNAs (sRNAs) play a central role in plant development and other important plant functions. Eukaryotic sRNAs are approximately 21-24-nucleotides molecules involved in many different cell processes, including development, heterochromatin formation, genome rearrangement, hormone signalling and metabolism [1]. There are different classes of sRNAs: short interfering RNAs, trans-acting RNAs and microRNAs (miRs) [1,2] amongst others.

miRs are small, endogenous RNAs that regulate gene expression in plants and animals by promoting cleavage or translation inhibition of mRNAs coded by specific target genes [3]. The stem-loop region of a long primary nuclear transcript (called miR precursor or pre-miR) is processed into 21-nucleotide RNAs by a multistep process involving the activity of DCL1 [4,5], HEN1 and HYL1 proteins [6,7]. AGO1 is the most important Argonaute protein in the plant miR pathway and preferentially binds small RNAs with a 5' terminal uridine such as most miRs [8-11]. miRs are involved in plant development, signal transduction, transcription factor accumulation, protein degradation, response to environmental stresses and pathogen invasion [12,13]. miRs are expressed at variable levels in diverse tissues and developmental stages [14,15], regulate their own biogenesis [16-18] and it has been reported that modest changes in miR level can result in substantial changes in the accumulation of mRNAs target genes [12,19]. These facts evidence that miR expression is under a tight and fine regulation.

Over-expression of miR genes or viral proteins, such as post-transcriptional gene silencing (PTGS) suppressors, cause multiple developmental defects by interfering with miR-guided target cleavage/degradation [20-22]. Viral infections also cause miR alteration and development abnormalities or symptoms [20,21,23-26]. However, it is not totally clear how viral infections interfere with miR pathways [25,27] and which are the consequences of such interference. In *Brassica sp*. for example, it has been reported that *Turnip mosaic virus *infection specifically induced the accumulation of miR1885 that targets a TIR-NBS-LRR class disease-resistant transcripts for cleavage [28]. These data clearly suggest an important role of miRs in host-pathogen interactions. Basically, miR pathways could be affected at transcriptional or post-transcriptional levels, the latter involving miRs processing, accumulation and activity. Most of the articles reporting miR alteration upon viral infection or transgenic expression of viral proteins uncovered post-transcriptional regulation involving the silencing suppressors activity [20,27,29]. Nonetheless it was also shown that expression of viral proteins with non-PTGS suppressor activity can also alter miRs accumulation [23]. To the best of our knowledge there are so far no reports of the alteration of miRs transcription upon plant viral infections.

In this work, we analyzed whether the transcriptional regulation of a miR promoter was altered by a plant virus infection. We selected miR164 since its accumulation is increased after *Tobacco mosaic virus *(TMV) infection [23,30], it is involved in plant development and its mRNA targets are well known [12,31-36]. miR164 is potentially transcribed from three independent loci, miR164a, miR164b and miR164c [17,37] and negatively regulates transcription factors with NAC domains such as CUC1 and CUC2 [12,31-36]. These factors are redundantly involved in the initiation of the shoot apical meristem and in the establishment of cotyledon and floral organ boundaries [9,13,38]. Recently, it was also shown that miR164 participate in a trifurcate feed-forward pathway involved in cell death in *Arabidopsis *leaves [19]. Here, we cloned the putative *Arabidopsis thaliana *miR164a promoter (P-miR164a), obtained *A. thaliana *transgenic lines expressing the *uidA *reporter gene (GUS) under its regulation, and studied its spatial and temporal expression. Finally, we analyzed the P-miR164a activity and the mature miR164, pre-miR164a and CUCs mRNAs accumulation after viral infections and hormone treatments.

## Results

### Bioinformatic analysis and cloning of the putative promoter sequence of the MIR164a gene

**Figure 1:**
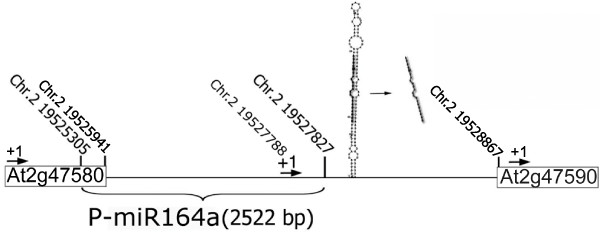
**Schematic representation of *Arabidopsis *miR164a locus and miR164a precursor**. Size and position of the miR164a putative promoter (P-miR164a) are indicated and mature miR164a highlighted. The rectangular boxes show the two flanking ORFs and their chromosome position. +1 indicates the putative transcription start sites. Arrows indicate the direction of transcription.

In order to characterize and define the proper miR164a gene promoter sequence and its regulatory elements, we performed an *in silico *analysis of the approximately 2.5 Kbp region located upstream of the mature miR164a sequence. A previous report showed that a 2.1 Kbp fragment upstream of miR164a is able to rescue null miR164 mutant lines [33]. Using the PlantCARE database http://bioinformatics.psb.ugent.be/webtools/plantcare/html/[39] we identified the putative transcription start site and promoter elements within the 2.5 Kbp. *In silico *analysis identified putative sequence elements related to stress response and others involved in gibberellic, abscisic, salicylic and jasmonic acids responses (Table 1). In addition, circadian control and anaerobic drought responses motifs were also predicted within this fragment and finally, 28 enhancer elements and 23 light-responsive related sequences were found not randomly distributed (see Additional File 1: Table S1 and Figure S1). A 2522 bp fragment (-2483 to +39, considering as +1 the transcription start site) was PCR amplified, cloned and completely sequenced to verify its identity and will be referred from now on as the miR164a promoter (P-miR164a). The miR164a locus within its genomic context and the miR164a precursor are represented in Figure 1.

**Table 1:** Putative *cis*-acting regulatory motifs in P-miR164a promoter.

TF	Site Name	First Organism Described	Position	Strand	sequence
**ABA**	ABRE	*Arabidopsis thaliana*	-565	+	TACGTG
		*Hordeum vulgare*	-567	+	CGTACGTGCA
		*Arabidopsis thaliana*	-1067	-	TACGTG
		*Arabidopsis thaliana*	-1069	-	TACGTGTC
		*Arabidopsis thaliana*	-1755	-	TACGTG

**Defense and Stress**	TC-rich repeats	*Nicotiana tabacum*	-597	-	ATTCTCTAAC

**Fungal Elicitor**	Box-W1	*Petroselinum crispum*	-495	+	TTGACC

**Gibberellin**	P-box	*Oryza sativa*	-499	+	CCTTTTG

**Jasmonic acid**	CGTCA-motif	*Hordeum vulgare*	-515	-	CGTCA
		*Hordeum vulgare*	-1217	+	CGTCA
		*Hordeum vulgare*	-1532	+	CGTCA
	
	TGACG-motif	*Hordeum vulgare*	-515	+	TGACG
		*Hordeum vulgare*	-1217	-	TGACG
		*Hordeum vulgare*	-1532	-	TGACG

**Salicylic acid**	TCA-element	*Brassica oleracea*	-547	-	GAGAAGAATA

Putative motifs recognized by transcription factors (TF) related to ABA, defense and stress, fungal elicitors, gibberellins, jasmonic acid and salicylic acid were detected using PlantCare program (Selected Matrix score for all elements ≥ 5). The site name, consensus sequence and the first organism where it was described were indicated. The positions were assigned relative to the miR164a transcription start site. (+) and (-) indicate sense or antisense DNA strands.

### P-miR164a is mainly expressed in the plant vascular tissue and its activity is developmentally regulated

**Figure 2:**
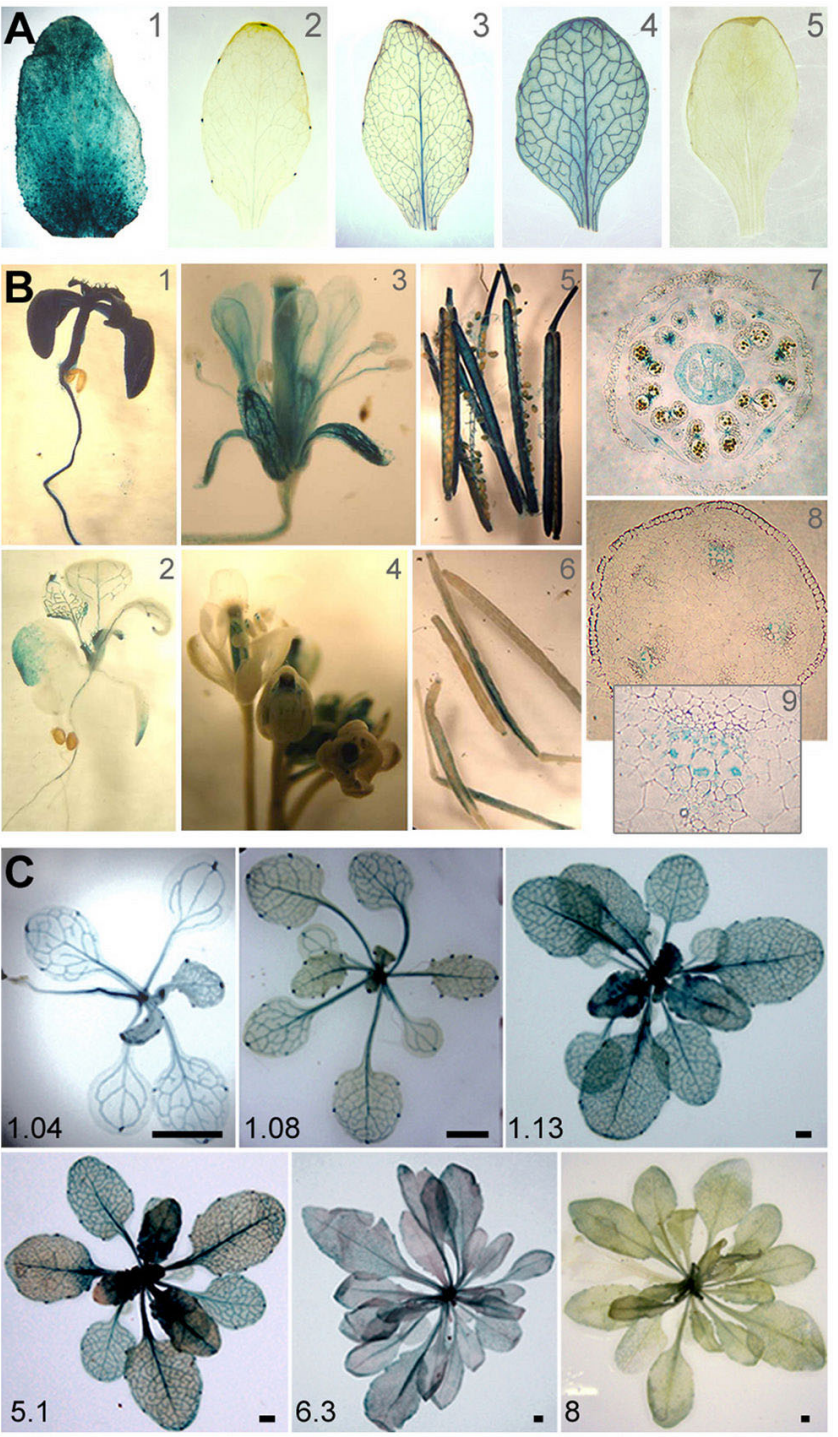
**Spatial and temporal expression patterns of GUS reporter gene driven by P-miR164a in transgenic *Arabidopsis *plants**. (A) Leafs from 4 week old plants of the different lines used for this study. A1: Control 35S::GUS transgenic *Arabidopsis *line. A2, A3 and A4: three independent P-miR164a::GUS transgenic *Arabidopsis *lines with showing low, intermediate or strong GUS activity (lines L35, L50 and L56 respectively). A5: Control EV::GUS transgenic Arabidopsis line where no GUS staining was detected. (B) GUS staining of plants, organs or sections of the control 35S::GUS transgenic *Arabidopsis *line (B1, B3 and B5) and P-miR164a::GUS L56 transgenic plants (B2, B4, B6 to B9). (B2) Staining leafs of one week-old plants. (B4) Mature and immature flowers. (B6) Detail of dehiscence zone of the siliques. (B7) Flower transverse section showing the reporter gene activity in the septum that divides both locus from each theca. (B8 and B9) Stem transverse sections with GUS staining found in developing xylem vessels. (C) Time course of P-miR164a transcription activity during the development of P-miR164a::GUS L56 transgenic plants. The plants were stained from stages 1.04 to stage 8. The most intense GUS staining was observed in stages 1.13 to 5.1. Bar = 0.5 cm.

In order to study the transcriptional activity of P-miR164a, we produced a set of *Arabidopsis *transgenic plants expressing GUS under its regulation (P-miR164a::GUS construct). As positive and negative controls, transgenic plants harboring a construct containing GUS controlled by the 35S *Cauliflower mosaic virus *promoter (35S::GUS), and transgenic plants for GUS lacking a regulatory sequence (EV::GUS, EV = empty vector) were obtained. Three P-miR164::GUS lines were selected to illustrate low (L35), medium (L50) and high (L56) levels of GUS expression out of 65 independent transgenic lines. All of them clearly showed a similar spatial pattern of expression (Figure 2A2, A3, and 2A4 respectively). The three lines segregated in a 3:1 ratio in T2 indicating a single locus of transgene insertion. In addition, one representative 35S::GUS line and one EV::GUS line were selected among several independent lines (Figure 2A1 and 2A5). The selected lines were brought to homozygosis, and the presence of 35S promoter, P-miR164 and GUS sequences was confirmed by PCR using specific primers (see Additional File 1: Figure S2).

Temporal and spatial GUS activity was observed in the different transgenic lines; GUS activity was detected in the entire plant vasculature (Figure 2A2-4, and 2B2) and in leaf hydathodes (Figure 2A2-4 and 2C) as previously described [33]. In reproductive organs, GUS staining was found in all carpel compound tissues and was stronger in its vasculature (Figure 2B4 and 2B7). GUS expression was also detected in siliques (Figure 2B6), petals and stamen vascular tissue and in the septum that separates the lobes of the each anther's thecae (Figure 2B7) whereas no GUS staining was found in the sepals. In detail, in stems, GUS stain was shown to be restricted to developing xylem vessels (Figure 2B8 and 2B9). To study the activity of P-miR164a during plant development, a time course assay was performed. Results revealed that all P-miR164::GUS transgenic lines had detectable GUS staining from seedlings up to almost stage 6.3 according to Boyes et. al., [40], showing a clear increase in the expression level at stages 1.12 to 5.1 (Figure 2C), while stage 8 had almost undetectable GUS activities. All these data suggested a developmental transcriptional regulation of P-miR164a during plant life cycle.

We additionally evaluated P-miR164a activity in different plant species by microprojectile bombardment or agro-infiltration assays. Promoter activity showed to be ubiquitous since it was conspicuous within monocotyledonous and dicotyledonous plants, such as *Allium cepa*, *Solanum tuberosum*, *Helianthus annuus *and *Nicotiana benthamiana *(see Additional File 1: Figure S3). In contrast, P-miR164a activity was not detected when transfecting mammalian BHK or insect Sf9 cells with appropriate constructs (see Additional File 1: Figure S4).

### Viral infections induce P-miR164a activity

**Figure 3:**
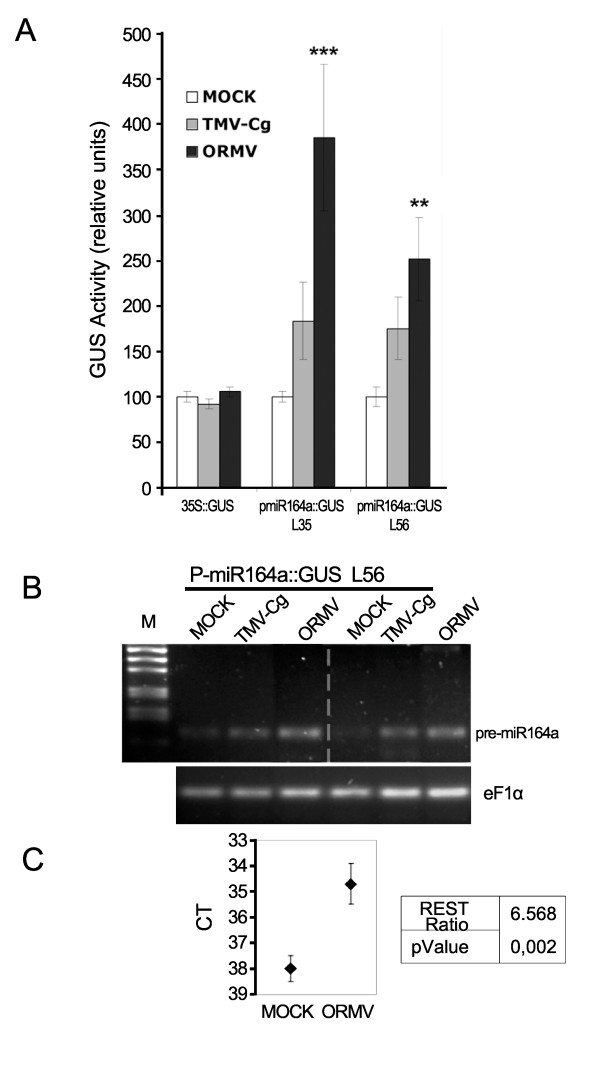
**Effects of virus infections on P-miR164a activity**. L56 and L35 P-miR164a::GUSArabidopsis transgenic lines and 35S::GUS control transgenic plants were virus-inoculated to quantify the effects in P-miR164a activity. (A) The bar chart shows the GUS activity mean value and standard error (SE) obtained in each group with n ≥ 10 from at least two biological replicates. Values were normalized to mock-inoculated controls of each line. Statistical comparisons were made by Kruskal-Wallis test with Dunn's post-test. Statistical differences between treated and mock-treated groups are shown. **p < 0.05, ***p < 0.01 compared to mock controls. (B) Representative RT-PCR of the pre-miR164a transcript in L56 transgenic plants. The housekeeping EF1α gene was amplified as an internal control. (C) Quantitative RT-PCR analysis to measure the level of the pre-miR164a in *Arabidopsis thaliana *Col 0 plants after ORMV infection. The chart shows the normalized CTs ± SE for each condition and the expression ratio between them calculated with REST algorithm.

It has been shown that miR accumulation is altered after viral infection most likely at post-transcriptional level [23,26,28,30]. To study whether virus infection could also interfere with miR pathways at the transcriptional level, we quantified P-miR164a-derived GUS activity after viral infection. We independently inoculated (or mock-inoculated) two P-miR164a::GUS lines showing low (line L35) and high (line L56) GUS expression level to consider the influence of the genomic context of the T-DNA insertion, 35S::GUS and EV::GUS plants with *Oilseed rape mosaic virus *(ORMV) and TMV-Cg. These two viruses were chosen because they clearly differ on the severity of the symptoms they produce on *Arabidopsis *plants, very mild in the case of TMV-Cg and strong in the case of ORMV, even when both viruses are proposed to be strains of the same species of the *Tobamovirus *family [41,42]. Also importantly, tobamoviruses were reported to alter miRs levels in tobacco and *Arabidopsis *[23,30]. In the experimental conditions, both tobamoviruses infected a high percentage of plants (above 95%) and accumulated to high titers (data not shown). First, the tissue localization pattern of GUS activity after viral infections was compared through histochemical staining assays and no clear alterations were detected upon infections (data not shown). Next, GUS activity was measured using a total rosette protein extract to minimize the characteristic patchy tissue distribution effect of areas with different infection levels. As shown in Figure 3A, GUS activity was statistically significantly increased after infection with the most severe virus (ORMV) in both P-miR164a::GUS lines. Even though not statistically significant, mean GUS activity values were also higher in both P-miR164a::GUS lines after TMV-Cg infection. As expected, GUS activity did not change in control 35S::GUS plants evidencing the specificity of P-miR164a induction upon virus infection.

To provide additional evidence that the transcriptional activity of P-miR164a is induced after infection, the level of pre-miR164a transcripts was analyzed by RT-PCR after viral infection. Figure 3B shows a clear increase of pre-miR164a accumulation after ORMV infection and a slight increase after TMV-Cg infection compared to mock-inoculated treatments in two biological replicates using L56 plants. This assay was repeated with similar results in line L35 (data not shown). Although all transgenic lines were equivalent to Arabidopsis wild type (Col-0) for this purpose (measuring endogenous pre-miR164a), tissues from L56 and L35 lines were analyzed to preserve the same genetic background used in GUS activity assays. In order to quantify the effect of virus infection on pre-miR164a abundance, qRT-PCR analysis was performed in *Arabidopsis *wild type plants after ORMV infection and compared to the mock-treated plants. Pre-miR164a gene expression increased more than six fold after ORMV infection, estimating gene expression ratio with a p-value of 0.005 through the REST algorithm [43] (Figure 3C).

Altogether, these data indicated that viral infections elevated the activity of P-miR164a evidencing that they also interfered with miRs pathways at the transcriptional level and that this induction was stronger in the case of ORMV, the most severe virus.

### The accumulation of miR164 and its target genes mRNAs are altered after virus infection

**Figure 4:**
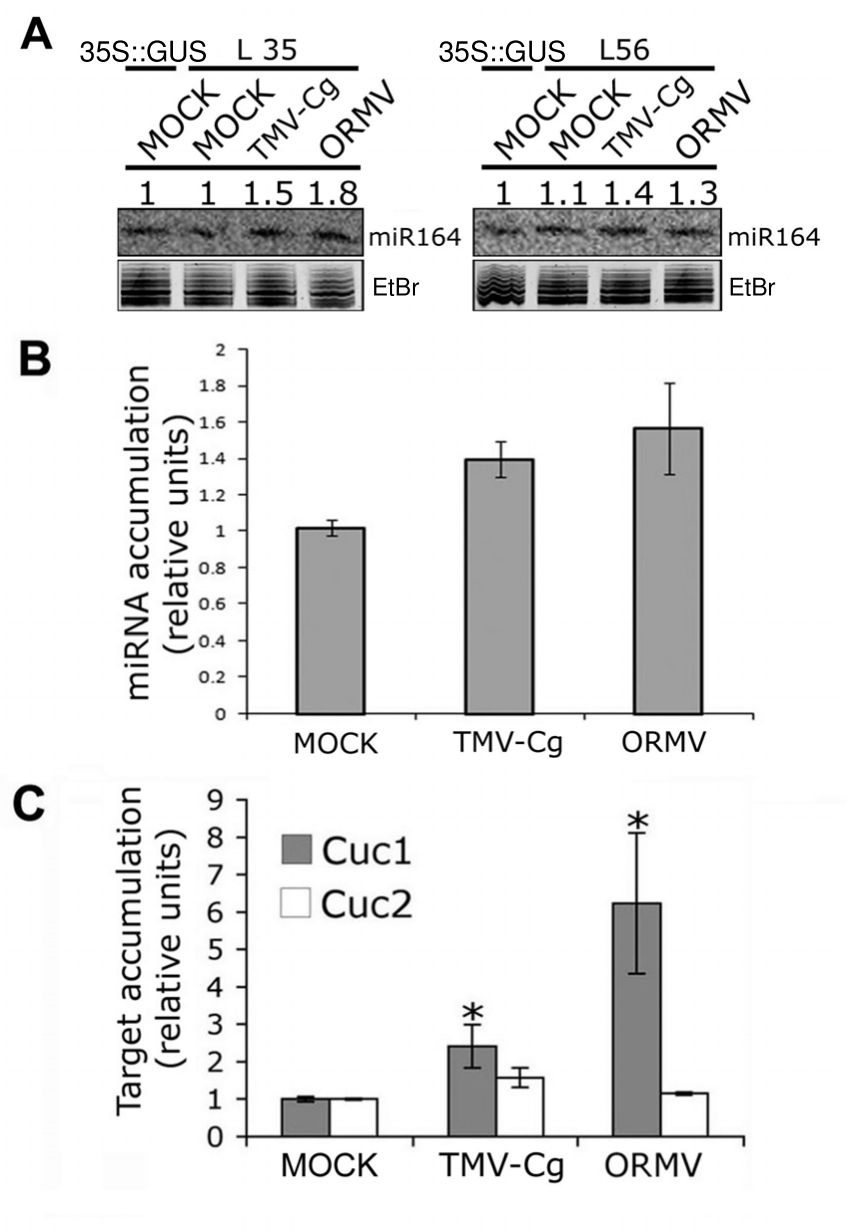
**Effects of TMV-Cg and ORMV infections on the accumulation of miR164 and CUC1 and CUC2 mRNAs**. (A) Northern blot analysis detecting the accumulation of miR164 in transgenic lines L35 and L56 after virus infection. Ethidium-bromide-stained rRNAs shown below each blot were used for data normalization. miR accumulation data were set relative according to the accumulation in mock-inoculated plants that was set at 1. (B) Average of two to four independent measurements of miR164 accumulation in L56 and L35 after virus infection. (C) CUC1 and CUC2 mRNAs transcript abundance determined by qRT-PCR and expressed in arbitrary units normalized to EF1α amount after virus infection. CUC1 and CUC2 transcript levels were computed relative to the levels in mock-inoculated plants that were set at 1. Each value represents the mean of four biological replicates. Bars indicate standard errors. (*) indicates a statistically significant difference (p < 0.008) for CUC1 relative expression in TMV-Cg and ORMV-infected plants compared to controls ones.

Next, we analyzed whether the induction of P-miR164a::GUS by virus infection also correlated with the levels of mature miR164 and its mRNA targets. The accumulation of mature miR164 in infected and mock-treated plants was detected and quantified by Northern-blot analysis. The hybridization with a miR164 probe was measured using a radioactivity-scanning device and normalized based on the amount of ethidium bromide-stained rRNA. The amount of miR164 in mock-treated plants was arbitrarily set as 1.0, and the rest of the data were computed relatively to these plants. As previously, L35 and L56 transgenic lines tissues were used to maintain the genetic background even though endogenous miRs were quantified. Figure 4A shows miR164 accumulation from two biological replicates of mock-treated plants (mock), TMV-Cg, and ORMV-infected plants. Figure 4B shows the mean values of miR164 quantification of two to four biological replicas, including the data shown on panel A. miR164 accumulation increased after infection with both tobamoviruses. The higher miR164 accumulation after infection might be due, at least partially, to the increase in P-miR164a transcriptional activity (as shown Figure 3A) since miR164b/c might also contribute to this observation.

Finally the effect of virus infection on miR164 activity was analyzed by measuring miR target accumulation by qRT-PCR using sets of primers annealing at both sides of the miR recognition site in order to only detect complete uncut mRNA targets (Figure 4C). Even though, no changes in CUC2 mRNA levels were detected after infections, CUC1 mRNA accumulated to higher levels in plants infected with both tobamoviruses (particularly with ORMV).

In conclusion, even though there was an induction of P-miR164a expression and pre-miR164a and miR164 accumulation upon infection, the mRNA levels of CUC1 mRNA target were also raised [44]. These results suggest that, in spite of the P-miR164a transcriptional induction, the viral infection caused a reduction of miR164 activity as a final outcome.

### Effects of hormone treatments on P-miR164a expression

**Figure 5:**
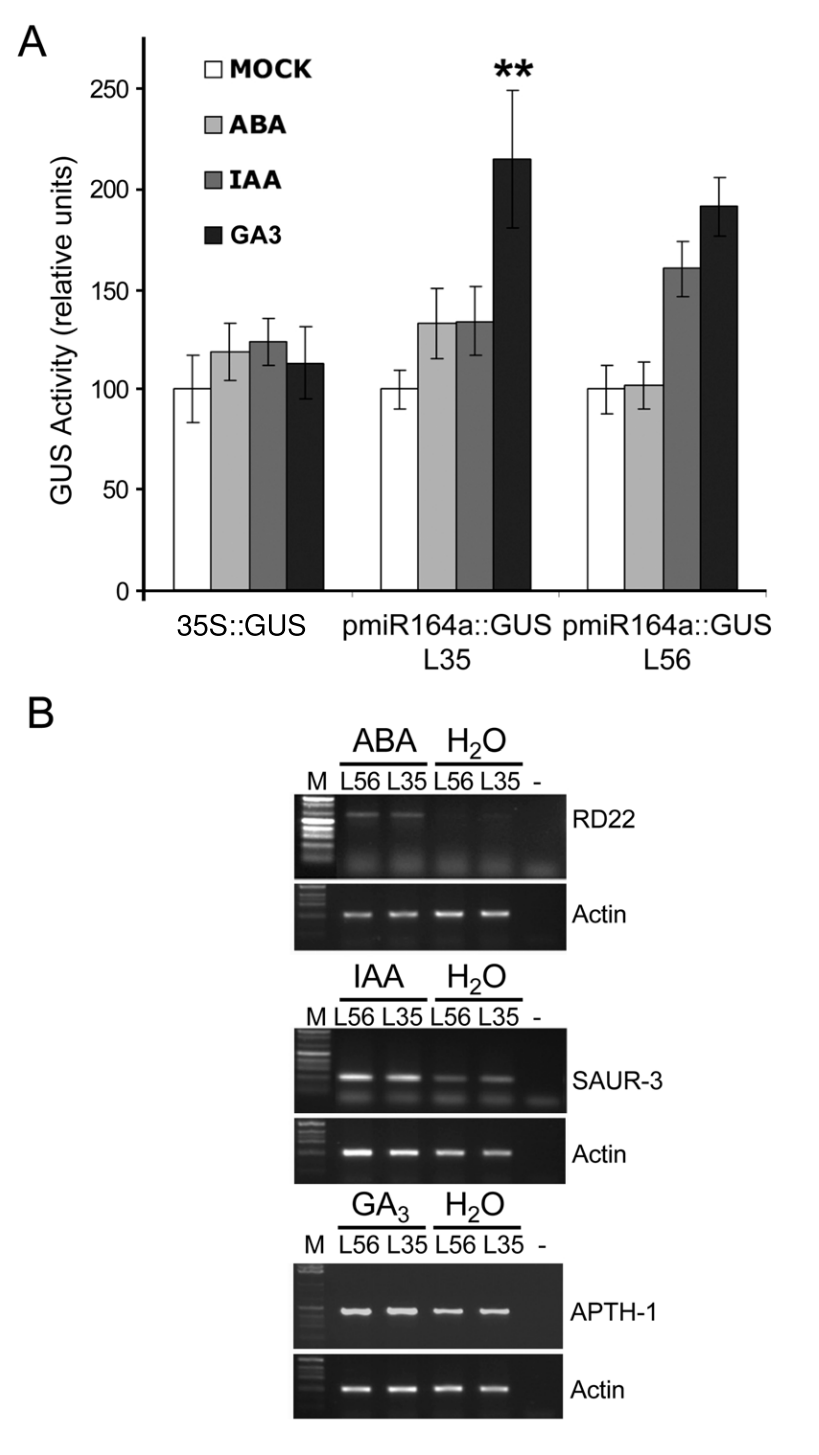
**Effects of hormone treatments in P-miR164a activity in transgenic *Arabidopsis *plants**. (A) L56, and L35 P-miR164a::GUS *Arabidopsis *transgenic lines and 35S::GUS control transgenic plants were hormone-treated as indicated in methods section. The bar chart shows the GUS activity mean value and the standard errors of results obtained in each group with n ≥ 10 from at least two biological replicates. Values were normalized to mock-treated controls of each line. Statistical comparisons were made by the Kruskal-Wallis test with Dunn's post-test. Statistical differences between treated and mock-treated groups are shown. **p < 0.05. (B) Effectiveness of hormone treatments by amplifying mRNAs that are known to be hormone inducible as ABA inducible RD22 (NM_122472); IAA inducible SAUR-AC1 (S70188) and GA3 inducible APT1 (NM_179383) genes. ACTIN2 gene was also amplified as internal control. M: 1 Kb DNA molecular marker; (-) Negative PCR control (without DNA).

Virus infections were reported to alter the concentration of phytohormones such as auxin, gibberellin and abscisic acid (ABA) [45,46]. As *in silico *analysis identified putative gibberellin and ABA responsive consensus elements within P-miR164a (Table 1), we analyzed whether P-miR164a activity changed after hormone treatments. P-miR164a::GUS transgenic lines (L35 and L56) and control 35S::GUS plants were sprayed with ABA, indole-acetic acid (IAA), or gibberellic acid (GA3) solutions as well as with water as a control. First, we determined that P-miR164a::GUS plants treated with hormones showed a GUS staining tissue pattern similar to that of mock-treated plants (data not shown). Figure 5A shows that GUS activity significantly increased after GA3 treatments in L35, while L56 showed a similar trend. No significant difference in P-miR164a activity was observed in plants upon exposure to ABA or IAA. As expected, GUS activity did not change in 35S::GUS plants after treatment, showing that hormone treatments could not induce this promoter. Effectiveness of all hormone treatments was confirmed by RT-PCR amplification of known hormone-responsive mRNAs (Figure 5B). Therefore, we concluded that GA3 treatment elevated the activity of P-miR164a promoter.

## Discussion

There is increasing information regarding the molecular events triggered after a plant virus infection including changes inplant gene expression, metabolism and development [27,47,48]. Some of these events may be required for the proper virus replication and spread, some may be plant responses and others may be just a side effect of virus infection. In turn, some of these alterations might be responsible for virus symptoms. Different molecules emerged as candidates to modulate this complex interaction, and a group of them are miRs [1,16,22,49,50]. Accordingly, miRs accumulation and activity were shown to be altered by virus infection and/or by the transgenic expression of viral proteins [20,21,23-26,30]. Different hypotheses, all of them involving post-transcriptional regulation, have been proposed [20,21,44,51,52]. Furthermore, this process may occur in the cytoplasm, after miR nuclear processing by DCL1 and subsequent nucleo-cytoplasmic transport [53].

In this work we showed for the first time that virus infections and GA3 treatment lead to enhanced transcriptional activity of P-miR164a thus revealing a novel mode of viral interference with plant miR biogenesis.

At early stages of leaf development, we showed that P-miR164a has a spatial expression pattern similar to the one reported by other authors [33,36]. Next, we further expanded the characterization to fully developed organs (Figure 2). One interesting observation was the identification of the highest P-mR164a activity on the vascular tissue of plants at stages 1.12 to 5.1 (Figure 2C). This time point correspond, according to Boyes et al, [40] just after the switch of the vegetative to the reproductive growth, and when several processes are initiated, including changes in hormone levels. This switch is also relevant for plant-virus interactions since it coincides with the time point when virus replication is transiently arrested, as reported by Lunello et al [54]. We also detected a strong reduction in P-miR164a activity at plant developmental stage 8.0 that correlated with a reported decrease in mature miR164 levels and an increase of its target gene *oresara-1 *(means "long-living" in Korean) mRNA (ORE-1), which positively regulates aging-induced cell death in leaves [19].

Upon ORMV infection, P-miR164a::GUS transgenic lines (L35 and L56) accumulated higher levels of GUS, showing that virus infection could directly or indirectly interfere with miR164a regulation at the transcriptional level (Figure 3A). Supporting these results, pre-miR164a accumulation also increased after viral infection in the same set of lines (Figure 3B). Nevertheless, the increased pre-miR164a accumulation could be as well explained by a change in the nuclear precursor rate processing. This possibility is unlikely in view of our GUS activity results although a partial contribution cannot be ruled out (Figure 3B, C). Furthermore, the ORMV infection elevated approximately by six fold the expression of the endogenous pre-miR164a compared to the mock-inoculated plants in wild type Col 0 plants (Figure 3C) also indicating that the transcriptional induction of P-miR164a is not affected by a genomic positional effect in the transgenic plants nor an artifact of the transgenic lines (Fig 3A, B, C). In sum, our results showed that miRs promoter activation should be considered to explain changes in miRs abundance during virus infection. Along this line, Csorva et al [51] demonstrated that tobamovirus infection increases miRs accumulation in *hst-15 *mutant plants (in which miR nuclear export is compromised) as well as in wild type plants. In this case, the increase in miR accumulation may be due to a transcriptional induction rather than a post-transcriptional regulation, given the fact that the PTGS suppressor and miRs are located in different cell compartments. Moreover this data is similar to the increase of miR transcription in response to different abiotic stresses reported by Liu et al [55].

Interestingly, our results show that the most severe tobamovirus, ORMV, significantly altered P-miR164a activity (in all lines evaluated) and produced a major increase in miR164 and in its target CUC1 mRNA accumulation (Figure 4). The fact that CUC1 (and not CUC2) was altered upon infection is in agreement with the observed degree of alteration of both target mRNAs in triple *miR164abc *mutant lines, since in rosette leaves CUC1 was the more responsive [36]. On the other hand, infection by a less severe virus such as TMV-Cg raised to a lesser extent (or did not change) P-miR164a activity and mature miR164 and CUC1 mRNA accumulation. These results evidence a correlation between infection severity and miRs pathways alteration. This agrees with a correlation recently reported between the increased accumulation of a set of selected miRs and symptom severity of tobacco plants separately infected with six different tobamoviruses (Bazzini et al, submitted) and, all together, this data may suggests a role of miRs alteration on symptom severity. Similar results were obtained in *Cucumber mosaic virus*/tomato interactions by Cillo et al. [56].

Even when we showed that virus infection elevates P-miR164a activity and increases pre-miR164a and mature miR164 accumulation, we detected higher levels of CUC1 mRNA target in rosettes leaves (Figure 4C). This reduction in miR activity is in agreement with reported data and was mostly explained by the action of viral PTGS suppressors [20,21]. *Tobamovirus *PTGS suppressors (p126k for TMV) mostly act by inhibiting the assembly of the RISC complex, although they cannot affect already sRNA-loaded RISC complexes as other stronger suppressors do [44,51,52]. Besides, their mode of action involves at least two functions: interference with sRNAs methylation and sRNAs binding [51,52]. This binding and sequestration of sRNAs as double-strand inactive forms is a common strategy of viral PTGS suppressors that might allow the stabilization and thus the increase of sRNAs accumulation and at same time reducing the miR activity level [51].

As it was mentioned before, phytohormones accumulation change after virus infection [45,46] and putative phytohormone-responsive elements were detected in the P-miR164a sequence by *in silico *analysis (Table 1). Consequently, hormones could be one of the candidate molecules mediating the linkage between viral infection and P-miR164a induction. In agreement, our data indicated that GA3 treatment induced P-miR164a promoter (Figure 5A). Additionally, Guo et al [12] reported that NAA treatment produces a modest induction of miR164 and a reduction of NAC1 target mRNA in *Arabidopsis *roots. Accordingly, it is reasonable to propose that miR promoter activity could be altered after viral infection by changes in phytohormones levels. Furthermore, several of the miRs whose accumulation are modified by tobamovirus infection were shown to be directly or indirectly related to phytohormone regulation (miR160 targeting ARF per example) or directly regulated by hormones [57,58]. Therefore, it makes sense to propose a crosstalk between hormone and miRs abundance alterations (or vice versa) after virus infection. In fact, recent work by Navarro et al [59,60] reported a link between miRs, hormones and pathogen resistance.

The mechanism of P-miR164 induction by virus infection and its implications are still unknown. The alteration of P-miR164 activity upon infection implies that virus infections mediate a nuclear modification but, as there are no reports of tobamovirus encoded proteins with nuclear activity, this could be the result of an indirect effect. Similarly, it is known that TMV infection causes a change in the nuclear localization of a putative regulator of auxin response involved in plant development that in turn alters auxin-mediated gene regulation [61-63]. We cannot rule out the existence of a feedback regulation of P-miR164a activity mediated by CUC1 target mRNA abundance. As previously mentioned, viral infection could decrease miR activity by its PTGS suppressors, increasing the miR-targets level. Consequently, P-miR164a might be induced to produce more miR to restore target accumulation. However, since the observed outcome was a higher level of miR-target after infection this suggests that PTGS suppressor action was stronger (reducing miR activity) than the resulting outcome of P-miR elevation of the transcription level at the time point analyzed. Additional evidence is needed to address this point. Although the biological role of P-miR164a induction during virus infection is still unknown, the transcription component described here must be taken into account when exploring the miR role in host-pathogen interactions.

## Conclusion

In conclusion, our work showed for the first time that, in addition the already described post-transcriptional effects, virus infection can interfere with miRs pathways at a transcriptional level. Further experiments are required to establish which proportion of the induced miR164 accumulation is due to the transcriptional effect, which is the precise mechanism involved and to uncover which is the biological relevance of this transcriptional component.

## Methods

### Constructs and transgenic plants

To obtain the P-miR164a::GUS and an empty equivalent construct (EV::GUS), a 2522-bp fragment upstream of the fold-back structure of miR164a (AT2G47585) was amplified from genomic *Arabidopsis thaliana *ecotype Col-0 DNA using specific P- MIR164a sense (containing a PstI tail) and antisense primers. The amplified fragment was cloned into pGEM-T Easy (Promega) and sequenced to confirm identity and integrity. The insert was then excised with EcoRI and cloned into the EcoRI site of pAKK1431 upstream of the *uidA *gene producing P-miR164a::*uidA *and antiP-miR164a::*uidA*. The orientation of the insert was checked, and a sense and an antisense version of the resulting recombinant intermediate plasmid were digested with PstI enzyme. The insert was subcloned into the PstI site of pCambia2300 http://www.cambia.org/daisy/cambia/585.html, giving rise to P-miR164a::GUS and EV::GUS, respectively. All constructs were electroporated into GV3101 *Agrobacterium tumefaciens *strain. *Arabidopsis thaliana *(Col-0 ecotype) was transformed by using the floral dip method [64] and the selected transgenic plants were confirmed by PCR using specific primers: Promo164-300 and INTRO AKK (for transgenic P-miR164a::GUS plants), 35S and INTRO AKK (for transgenic 35S::GUS plants), and GUS up and GUS low (for all transgenic lines). In addition, a PCR amplification of *Arabidopsis *Actin-2 (NM_112764) gene was performed as an internal control by using primers Actin-2 up and Actin-2 low. All primers are listed in Additional File 1: Table S2.

### β-Glucuronidase (GUS) histochemical and fluorometric assessments

Qualitative β-glucuronidase (GUS) histochemical and quantitative fluorometric assays were performed as reported [65]. X-glu (5-bromo-4-chloro-3-indolyl-glucuronic acid, Inalco S.P.A., Milano, Italy) or MUG (β-D-glucoronide hydrate, Fluka, BioChemika, UK) were used as substrates. For fluorometric assessments, the technique was adapted to an automatic measurement of real-time enzymatic activity in a 96-well microplate and fluorescence was measured on a SpectraMax^® ^GEMINI EM spectrofluorometer (Molecular Devices Corporation, Sunnyvale, CA, USA). Data were extracted using the SoftMax Pro 5 software.

### Plant material and virus infection assays

TMV-Cg and ORMV isolates were maintained in *A. thaliana *plants ecotype Col-0. Plants were grown in growing chambers (22°C, 16-8 h photoperiod and a light intensity of 100 μE m-2 s-1). Mock inoculated plants were buffer-rubbed. Sampling was done at 7 days after inoculation (in the case of plants treated with ORMV) and at 9 days after inoculation (in the case of plants treated with TMV-Cg). Plant infection was verified by ELISA using Agdia (RMV) and Bioreba (TMV) commercial kits.

### Hormone Treatments

*Arabidopsis *ecotype Colombia (Col-0) and T3 seedlings of transgenic *Arabidopsis *plants were grown in growing chambers (at 20-25°C, 8 h dark-16 h light cycle) for 4 to 5 weeks and used in hormone treatment experiments prior to bolting. Plants subjected to treatment were sprayed with 50 ml of 100 μM ABA, 100 μM indole-acetic acid (IAA), 50 μM gibberellic acid (GA3) or mock-treated with water and incubated for 6 h under dim light. Following, at least four plants of each line were histochemically stained for GUS detection [65], while twelve plants of each line were immediately frozen in liquid nitrogen and stored at -80°C until RNA isolation or GUS activity quantitative analysis [65]. At least two independent assays were performed on P-miR164a::GUS lines (L35, L56), 35S::GUS and EV::GUS transgenic lines. Averages were calculated after data normalization to mock-treated plants. Effectiveness of hormone treatments was confirmed by RT-PCR using the following *Arabidopsis thaliana *genes: GA3 inducible APT1 (NM_179383) [66], ABA inducible RD22 (NM_122472)) [67] and IAA inducible SAUR-AC1 (S70188) [68]. Actin-2 gene was also amplified as a control (see Additional File 1: Table S2 for primers sequence).

### Statistical analysis

Statistical comparisons of relative GUS activity among plant groups were performed by Kruskal-Wallis test with Dunn's post-test (GraphPad Prism 5; GraphPad Software, http://www.graphpad.com/ and InfoStat software (*InfoStat version 2008*. Grupo InfoStat, FCA, Universidad Nacional de Córdoba, Argentina) was employed.

### miR analysis

miRs were isolated from pools of at least three *Arabidopsis *rosette leaves using the miRVana Kit (Ambion. USA) and then, quantified measuring absorbance at 260 nm using a spectrophotometer (NanoDropTechnologies). All RNA samples were adjusted to the same concentration to homogenize the miR input and 20 micrograms of sRNA were resolved in 17% polyacrylamide gels containing 7 M urea. After electrophoresis, RNA was blotted to GeneScreen Plus membrane (PerkinElmer Life Science, USA). Probes homologous to *Arabidopsis *miR164 were end-labelled using [γ^32^P] ATP and PNKinase. The probe was purified from the unincorporated label with Qiaquick Nucleotide Removal kit (QIAGEN). The eluted radiolabeled oligo was incubated with the membrane in 3× SSC, 5% SDS and 10× Denhardt's solution at 50°C overnight. The membrane was washed 2 times with the same solution buffer for more than 30 minutes and exposed for one night. The intensity of each band was quantified by using a Typhoon Trio (Amersham Biosciences, USA). The Typhoon Trio was also used to quantify the RNA loaded in each well by scanning the ethidium bromide stained gel previously to the transfer to the membrane. Data from these analyses were used to normalize the radioactivity intensity of each band, based on the total sRNA loaded in each well. The value for the miR species in mock-treated plants was set as 1.0 and the other data were calculated relative to this value.

### Quantitative real-time polymerase chain reaction

Total RNA was isolated from pools of rosette leaves of three *Arabidopsis *plants using the RNeasy Plant Mini Kit (Qiagen), quantified (NanoDropTechnologies) and treated with DNase I (Invitrogen). First-strand cDNA was synthesized using Superscript III (Invitrogen, USA), and oligo d(T)_20 _according to Superscript manufacturer's instructions (Invitrogen, USA). The oligonucleotide primer sets used for real-time qPCR analysis were designed using Primer Express 2.0 software (Applied Biosystems) to amplify a fragment containing the miR target recognition site. The primers are listed in Additional File 1: Table S2. Experiments were carried out using four biological replicates in an Applied Biosystems 7500 equipment. Arabidopsis elongation factor-1α (EF1α, NM_125432) was used as internal control. The mean values were calculated and the standard errors (± SE) were computed taking into account a primer efficiency correction [43]. For miRs targets quantification the statistically significant differences in expression between control and treatments samples were calculated using Kruskal-Wallis test using the InfoStat software (version 2008), where the cut-off was set to p < 0.05.

Detection of the 91 bp pre-miR164a fragment by RT-PCR was carried out using cDNA synthesized as described above and the primers listed in Additional File 1: Table S2. The PCR cycle used was the following: 94°C for 5 min followed by 35 cycles of 94°C 30 s; 60°C 30 s, 72°C 30 s.

## Authors' contributions

AAB conceived and designed the study, built all genetic constructs; carried out the expression in animal's cells, the RT-PCR assays to detect the miR164a precursor, supervised several assays and wrote the paper. NIA performed the bioinformatics analysis of the promoter region, carried out the hormone assays and participated in the histological analysis and aided with the writing. CAM performed the virus infection experiments, the GUS fluorometrics measurements and all the statistics analysis. VCM performed the histological analysis and participated in the bioinformatics analysis and discussion of the results. GC performed qRT-PCR to detect target genes. GAM performed ballistic assays on several plants species and edited all figures. MCR performed the molecular characterization of all transgenic lines. AJD performed the Northern blots to detect miR164. HEH analyzed data and discussed the results. MdV discussed the results and wrote the paper. SA coordinated, conceived and designed the study, participated in several assays discussed the results and wrote the paper. All authors read and approved the final manuscript.

## Supplementary Material

Additional file 1 **Supplemental material**. **Table S1**. Putative *cis*-acting regulatory motifs in P-miR164a other than the ones listed in Table 1. **Figure S1**. Schematic representation of all putative motifs recognized by transcription factors (TF) by the PlantCare program in P-miR164a (Selected Matrix score for all elements >= 5). **Figure S2**. Molecular characterization of the transgenic plants used along this work. **Figure S3**. Transient expression of P-miR164a::GUS in different plant species. **Figure S4**. Transient expression of P-miR164a::GUS in animal cells. **Table S2**. Primer sequences Click here for file
