# Supplementary material for: Virus infection elevates transcriptional activity of miR164a promoter in plants
Source: BMC Plant Biol. 2009 Dec 30;9:152. doi: 10.1186/1471-2229-9-152 (PMC2809068; doi:10.1186/1471-2229-9-152)
Supplement: Additional file 1 — Supplemental material. Table S1. Putative cis-acting regulatory motifs in P-miR164a other than the ones listed in Table 1. Figure S1. Schematic representation of all putative motifs recognized by transcription factors (TF) by the PlantCare program in P-miR164a (Selected Matrix score for all elements >= 5). Figure S2. Molecular characterization of the transgenic plants used along this work. Figure S3. Transient expression of P-miR164a::GUS in different plant species. Figure S4. Transient expression of P-miR164a::GUS in animal cells. Table S2. Primer sequences [file 1471-2229-9-152-S1.PDF]

Table S1:Transcription factors binding sequences and tested factors on micro 164a promoters

TF	Site Nam	First Organism Described	Position	Strand	Sequence
**AnaerobicInduction**	ARE	*Zea mays*	-1696	+	TGGTTT
				+	TGGTTT
**Circadian**	Circadia	*Solanum lycopersicum*	-1297	-	CAANNNNATC
			-1699	-	CAANNNNATC
			-2021	-	CAAAGATATC
**Drought**	MBS	*Arabidopsis thaliana*	-249	-	TAACTG
			-403	-	CGGTCA
			-1795	+	TAACTG
**EndospermExpression**	GCN4_moti	*Oryza sativa*	-1258	-	TGTGTCA
	Skn-1_moti		-1342	-	GTCAT
**Ethylene**	ERE	*Dianthus caryophyllus*	-1500	-	ATTTCAAA
**Light**	3-AF1 bindinsit	*Solanum tuberosum*	-860	-	TAAGAGAGGAA
	AE-bo		-1649	+	AGAAACAA
	ATCT-moti		-2436	+	AATCTAATCT
	Box		-42	+	ATTAAT
			-778	+	ATTAAT
			-1003	+	ATTAAT
			-1065	+	ATTAAT
			-1131	+	ATTAAT
			-1178	+	ATTAAT
			-1371	+	ATTAAT
			-1447	+	ATTAAT
	Box	*Pisum sativum*	-1500	-	TTTCAAA
	Box	*Pisum sativum*	-263	+	TTTCAAA
	Box I	*Nicotiana plumbaginifolia*	-984	-	GTGGATATTAT AT
	G-Box	*Daucus carota*	-880	+	TACGTG
		Antirrhinum majus	-880	-	CACGTA
		Solanum tuberosum	-1030	-	CACATGG
		Pisum sativum	-1032	-	CACACATGGAA
		Antirrhinum majus	-1382	+	CACGTA
		carota	-1382	-	TACGTG
		carota	-2070	-	TACGTG
		Antirrhinum majus	-2070	+	CACGTA
	GAG-motif Spinacia		-1281	-	AGAGATG
	I-bo		-762	+	CTCTTATGCT
	Sp		-954	-	CC(G/A)CCC

Table S1. Putative *cis*-acting regulatory motifs in P-miR164a other than the ones listed in Table 1.

Putative motifs recognized by transcription factors (TF) related with anaerobic induction, circadian oscillations, drought, endosperm expression, ethylene and light were found using PlantCare program (Selected Matrix score for all elements >= 5). The site name, consensus sequence and the first organism where it was described were indicated. The positions were assigned relative to the Mir164a transcription start site. (+) and (–) indicate sense or antisense DNA strands.

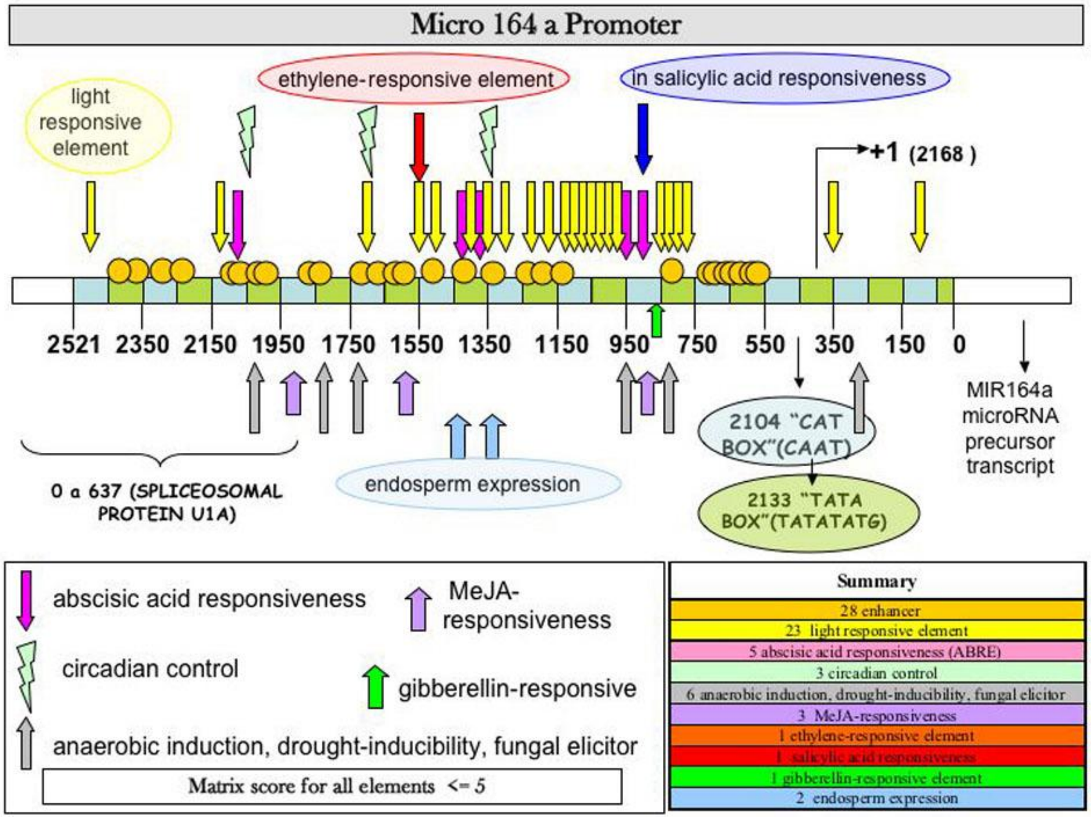

Figure S1. Schematic representation of all putative motifs recognized by transcription factors (TF) by the PlantCare program in P-miR164a (Selected Matrix score for all elements >=5).

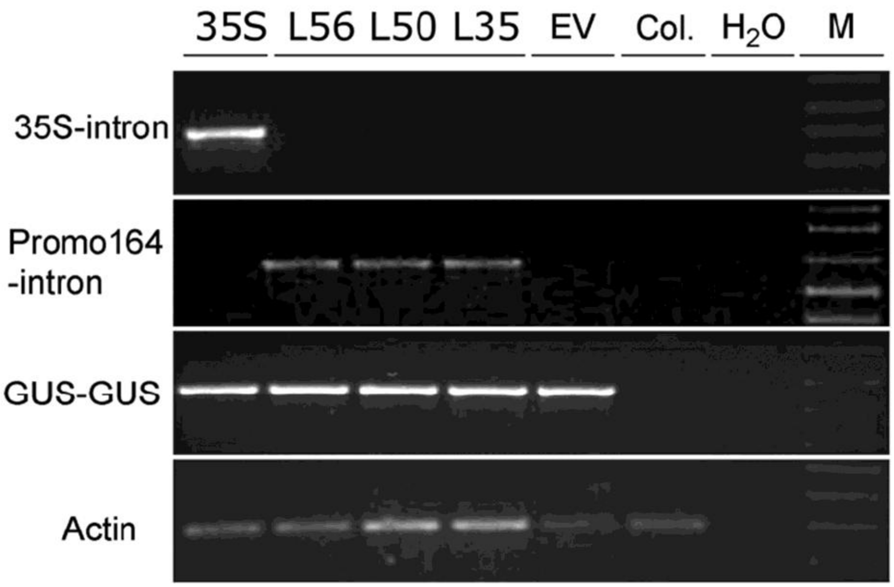

Figure S2. Molecular characterization of the transgenic plants used along this work.

PCR detection of a 35S-intron fragment from a 35S::GUS transgenic *Arabidopsis* line, Promo164-intron fragment from P-miR164a::GUS transgenic lines L56, L50 and L35, a; GUS fragment from all transgenic lines and an ACTIN2 fragment from all plants used. Non-transgenic (Col 0) was used as a negative control and PCR without DNA template (H_20) was used to check DNA contamination.

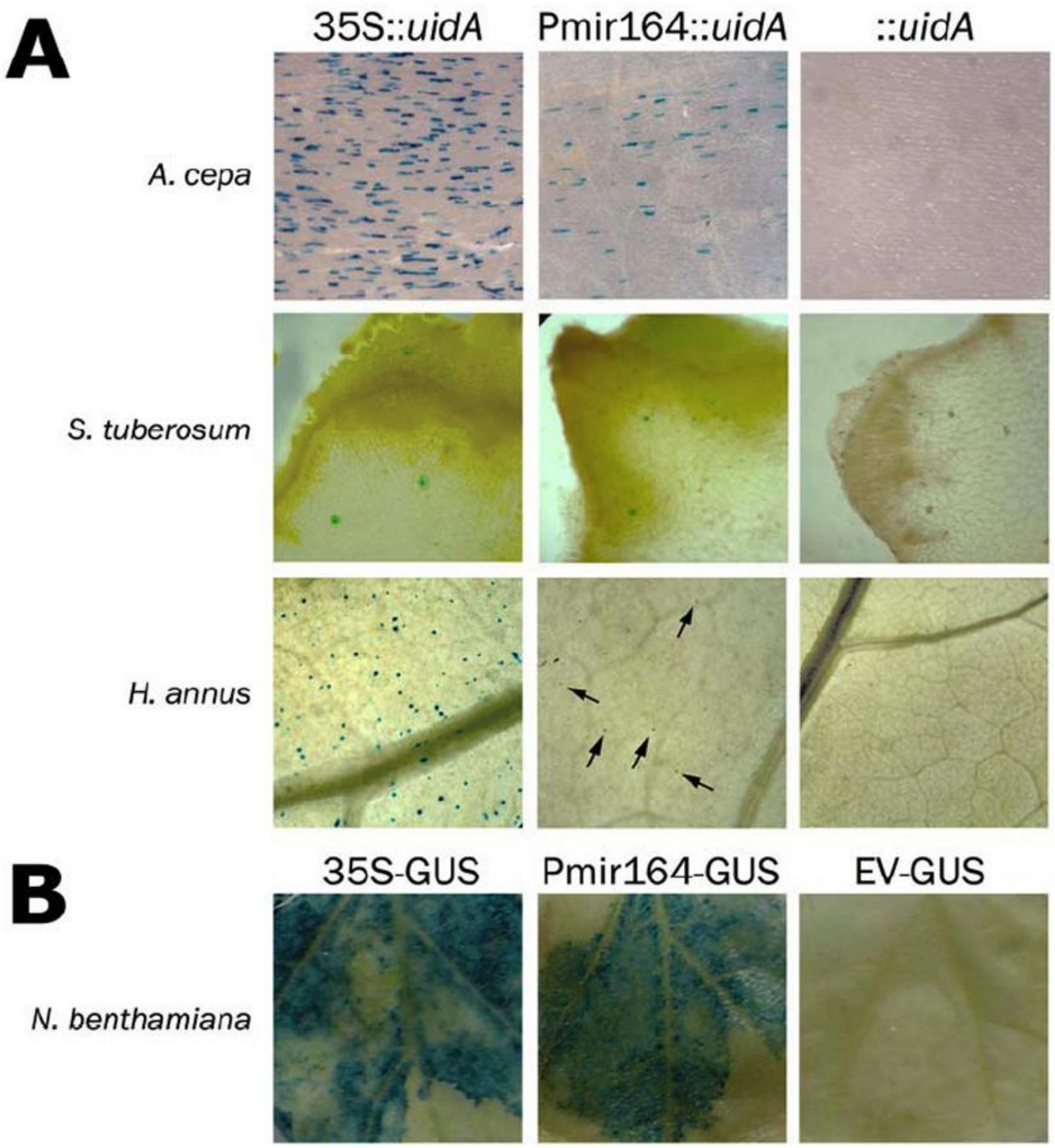

Figure S3. Transient expression of P-miR164a::GUS in different plant species.

(A) P-miR164a::*uidA* construct bombarded onto of *Allium cepa*, *Solamun tuberosum* and *Heliantus annus* tissues and detection of GUS reporter gene was performed. Arrows point to tiny blue transformed cells. 35S::GUS and EV::GUS constructs were used as positive and negative controls.

(B) *Nicotiana benthamiana* leaves agoinfiltrated with 35S::GUS; P-miR164a::GUS and EV::GUS constructs, and stained for GUS detection 3 days after infiltration.

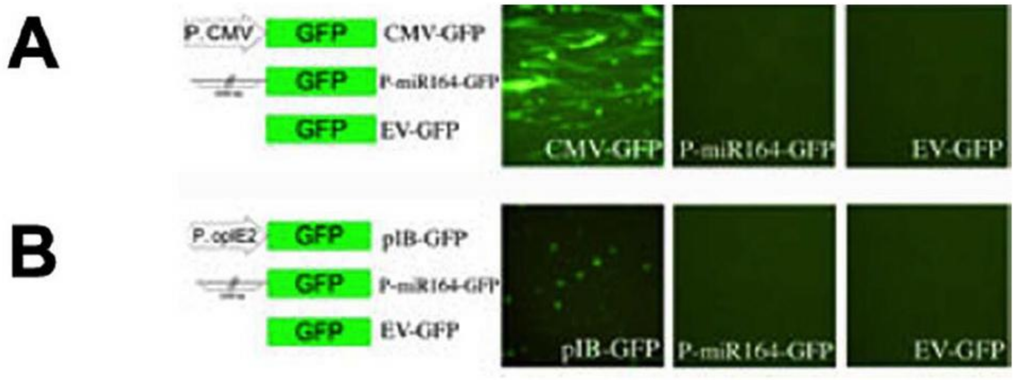

Figure S4. Transient expression of P-miR164a::GUS in animal cells.

(A) CMV promoter, P-miR164a and a non-regulatory fragment were cloned upstream of the green fluorescent protein gene. Mammalian BHK cells were transfected with each construct and GFP signal observed under a fluorescence microscope.

(B) Schematic constructs used for expression in insect Sf9 cells. *OpIE*2 promoter, P-miR164a and a non-regulatory fragment were cloned upstream of the green fluorescent protein gene. Insect cells were transfected with each construct and GFP signal observed under a fluorescence microscope.

Table S2. Primer sequences

Primer	Sequence (5'-3')
PMIR 164a sense	CTGCAGCATGCCCATGAACTCAGC
PMIR 164a antisense	CACCCGCATTTCCAAGCA
P164-300	AGTTCAACCCTTCTTTTGCGTG
INTRO AKK	GCAAACACCTGCTGAAACCTTT
35S	ATCTCCACTGACGTAAGGGA
GUS up	GATAGCGCGTGACAAAAACC
GUS low	GGGATCCAGGCCTTTGTTTGCCTCCCTGCTG
ACTIN-2 up	AACATTGTGCTCAGTGGTGG
ACTIN-2 low	TCATCATACTCGGCCTTGG
APT1 up	TCCCAGAATCGCTAAGATTGCC
APT1 low	CCTTTCCCTTAAGCTCTG
RD22 up	GTGGCTAAGAAGAACGCACCGATGC
RD22 low	CTCTAATTTTATTATAGGTTTTTGCAAAAC
SAUR-AC1 up	TTGAGGAGTTTCTTGGGTGC
SAUR-AC1 low	CATGGTATTGTTAAGCCGCC
Cuc1 up	TCTGCCGGTTCTGCAATTG
Cuc1 low	ATGAAAGCTCGCATCGGTATG
Cuc2 up	ATGAAAGCTCGCATCGGTATG
Cuc2 low	TAGCACCAACACAACCGTCACA
El1α up	ATTGGTAACGGTTACGCCC
El1α low	TCTCCTTACCAGAACGCCTGTC
PreMiR164aF	CCCTCATGTGCTTGGAAATG
PreMiR164aR	GCAAATGAGACGGATTTCGTG

List of primers used in this work.
